# Effects and Mechanism of Nano-Copper Exposure on Hepatic Cytochrome P450 Enzymes in Rats

**DOI:** 10.3390/ijms19072140

**Published:** 2018-07-23

**Authors:** Huaqiao Tang, Min Xu, Fei Shi, Gang Ye, Cheng Lv, Jie Luo, Ling Zhao, Yinglun Li

**Affiliations:** 1 Department of Pharmacy, School of Animal Medicine, Sichuan Agricultural University, Chengdu 611130, China; turtletang@163.com (H.T.); xumin010101@163.com (M.X.); shifei0202@163.com (F.S.); yegang010101@163.com (G.Y.); lvcheng010101@163.com (C.L.); luojie1588@sina.cn (J.L.); zhaoling010101@163.com (L.Z.); 2 School of Medicine, Tongren Polytechnic College, Guizhou 554300, China

**Keywords:** nano-copper, oral exposure, liver toxicity, CYP450 enzymes, mechanism

## Abstract

Although nano-copper is currently used extensively, the adverse effects on liver cytochrome P450 (CYP450) enzymes after oral exposure are not clear. In this study, we determined the effects and mechanisms of action of nano- and micro-copper on the expression and activity of CYP450 enzymes in rat liver. Rats were orally exposed to micro-copper (400 mg/kg), Cu ion (100 mg/kg), or nano-copper (100, 200 and 400 mg/kg) daily for seven consecutive days. Histopathological, inflammatory and oxidative stress were measured in the livers of all rats. The mRNA levels and activity of CYP450 enzymes, as well as the mRNA levels of select nuclear receptors, were determined. Exposure to nano-copper (400 mg/kg) induced significant oxidative stress and inflammation relative to the controls, indicated by increased levels of interleukin (IL)-2, IL-6, interferon (IFN)-γ, macrophage inflammatory protein (MIP-1), total antioxidant capacity (T-AOC), malondialdehyde (MDA), inducible nitric oxide synthase (iNOS) and nitric oxide (NO) after exposure. The levels of mRNA expression of pregnane X receptor (PXR), constitutive androstane receptor (CAR) and aryl hydrocarbon receptor (AHR) were significantly decreased in 400 mg/kg nano-copper treated rats. Nano-copper activated the expression of the NF-kappa B (NF-κB), mitogen-activated protein kinase (MAPK) and signal transducer and activator of transcription (STAT)3 signaling pathways. Nano-copper decreased the mRNA expression and activity of CYP 1A2, 2C11, 2D6, 2E1 and 3A4 in a dose-dependent manner. The adverse effects of micro-copper are less severe than those of nano-copper on the CYP450 enzymes of rats after oral exposure. Ingestion of large amounts of nano-copper in animals severely affects the drug metabolism of the liver by inhibiting the expression of various CYP450 enzymes, which increases the risk of drug-drug interactions in animals.

## 1. Introduction

Cu nanoparticles (Nano-copper) represent a promising modality in biomedical research and clinical applications. Nano-copper has shown great potential as an antibacterial agent and additive in animal feed [1,2,3]. Furthermore, nano-copper has been widely used in industry, e.g., as an additive in lubricants, for metallic coatings, and as a highly reactive catalyst in organic hydrogen reactions [4,5].

There are numerous data regarding the toxicity of copper compounds. Although the nanoparticles may primarily target respiratory organs, they may potentially infiltrate the gastrointestinal tract after introduction into the body [6,7,8]. The gastrointestinal system, liver, and kidneys are the main target organs of copper toxicity via oral exposure. The toxicity of Cu mainly manifests in drowsiness and anorexia, disruption of the epithelial lining of the gastrointestinal tract, hepatocellular necrosis, and acute tubular necrosis in the kidney [9,10].

The toxic effects of nano-copper have been shown to be dependent on particle size, shape, dosage, and, in particular, surface chemistry [11,12]. Most recently, studies have indicated that an overload of copper can induce a set of toxicological changes such as hepatocirrhosis, alteration of lipid biosynthesis gene expression, oxidative stress, renal dysfunction and alimentary canal in vivo [13,14,15]. Oxidative stress is always accompanied by inflammation [16,17], and nano-particles have been found to cause inflammation in the lungs [18,19]. However, there is little data on the ability of nano-particles to cause liver inflammation after oral administration. Oxidative stress and inflammation are the main factors in regulating the expression and activity of cytochrome P450 enzymes (CYP450) [20,21,22,23]. CYP450 isoenzymes are responsible for the metabolism of about 90% of drug oxidation reactions [24]. CYP450 inhibition or induction results in alteration of metabolism of therapeutically administered drugs, potentially leading to increased toxicity or diminished efficacy and treatment failure [25]. CYP3A4 acts as a predominant isoform in the liver for the metabolism of drugs [26]. Nuclear receptors pregnane X receptor (PXR) and constitutive androstane receptor (CAR) regulate the expression of the CYP450 3A and 2B isoforms, whereas CYP450 1A is induced by the aryl hydrocarbon receptor (AHR) [27]. In general, induction and suppression of expression are the causes of drug-drug interactions or xenobiotics-drug interactions [28,29,30].

In this study, we investigated the in vivo toxicity of nano-copper and micro-copper on CYP450 enzymes following a 7-day oral dose regimen in rats by evaluating the mRNA levels and activities of CYP450 enzymes in the liver. Oral administration was chosen because nano-copper is often given to animals orally [31,32]. Further, we investigated the changes in histopathology, inflammation, oxidative stress, and various signaling pathways in the liver to elucidate the mechanism of CYP450 expression change after exposure to nano-copper. In this work, we report for the first time, to the best of our knowledge, that nano-copper exerts significant changes to liver CYP450 expression in vivo, the extent of which may potentially interfere with disposal of many drugs administered to animals.

## 2. Results

### 2.1. Physiochemical Characterization of Nano- and Micro-Copper

**Figure 1:**
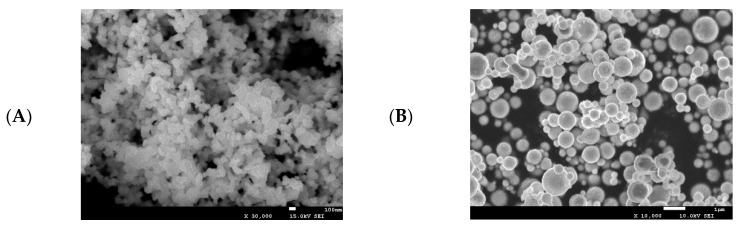

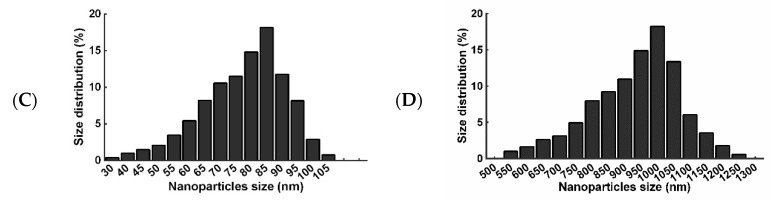
Physiochemical characterization of nano- and micro-copper. Nano-copper (**A**,**C**), micro-copper (**B**,**D**). The average particle diameters of nano- and micro-copper were 80 nm and 1 μm, respectively.

The physiochemical characteristics of nano- and micro-copper are shown in Figure 1. The morphology and actual size of nano-copper and micro-copper were characterized by scanning electron microscopy (SEM) and laser particle size analyzer. The morphology of nano- and micro-copper is spherical (Figure 1A,B), and the size distribution is rather wide (Figure 1C,D). The average particle diameters of nano- and micro-copper were 80 nm and 1 μm, respectively. The polydispersity index (PDI) of nano- and micro-copper were 0.34 and 0.46, respectively, according to the laser particle size analyzer test. The size and shape of the copper particles were consistent with the data provided by the manufacturer.

### 2.2. Histopathological Changes

**Figure 2:**
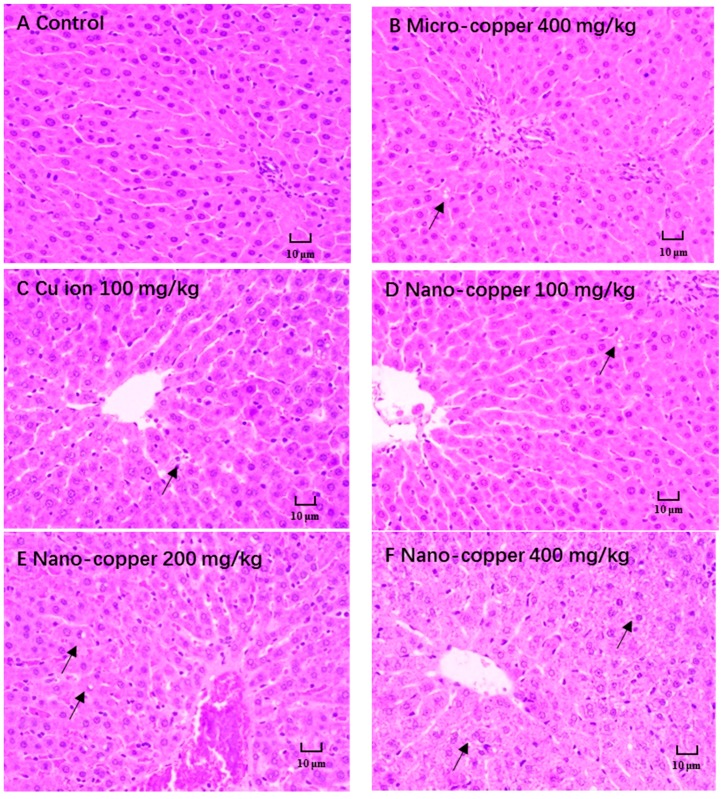
The histopathological changes of different copper treatments on rat liver. (**A**) Control group, (**B**) micro-copper treated group, (**C**) CuCl_2_·2H_2_O treated group, (**D**–**F**) nano-copper treated group. Lesion severity (→) increase with increasing doses of nano-copper.

Our findings confirmed the results of previous studies showing that a single or short-term oral exposure of nano-copper induces severe damage to the liver. The major histopathological change was degeneration of hepatocytes (→) (Figure 2). With high doses of nano-copper, lesions were more pronounced.

### 2.3. Inflammation

**Figure 3:**
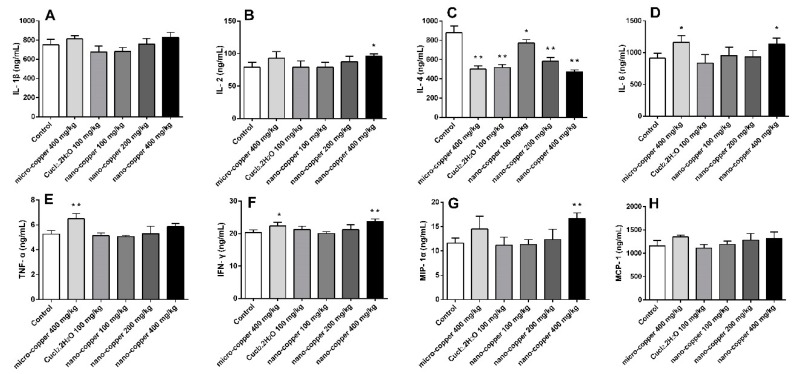
The levels of cytokines in copper treated rats. IL-1β (**A**), IL-2 (**B**), IL-4 (**C**), IL-6 (**D**), IFN-γ (**E**), TNF-α (**F**), MIP-1 (**G**), MCP-1 (**H**). * *p* < 0.05, ** *p* < 0.01 vs. control group. High dose of nano-copper significantly induced the level of cytokines in liver of rats. Micro-copper also induced the release of some of the cytokines, though the species of rat treated differed, and the Cu ion has no induction effect on cytokines.

Nano- and micro-copper induced the release of several cytokines (Figure 3). The levels of IL-2 and MIP-1 increased significantly in 400 mg/kg nano-copper treated rats. The levels of IL-6 and IFN-γ increased significantly in micro-copper and 400 mg/kg nano-copper treated rats. The levels of TNF-α were only induced by micro-copper. Finally, the levels of IL-4 decreased significantly in copper treated rats.

### 2.4. Oxidative Stress

**Figure 4:**
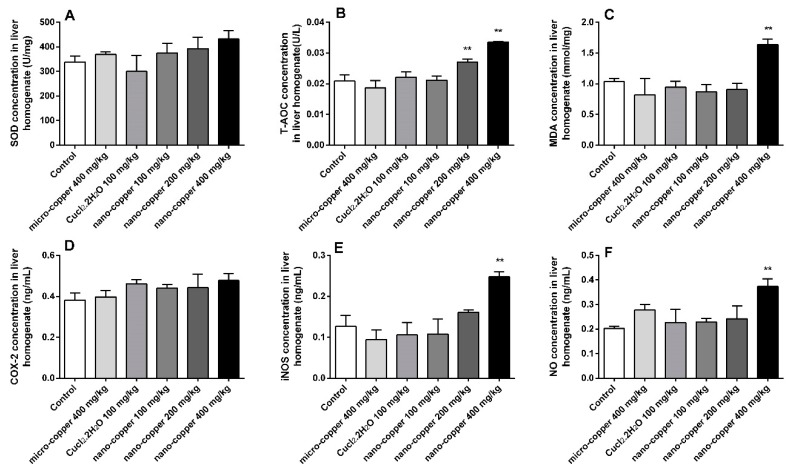
Oxidative index changes in rat livers. SOD (**A**), T-AOC (**B**), COX-2 (**C**), MDA (**D**), iNOS (**E**), NO (**F**). * *p* < 0.05, ** *p* < 0.01 vs. control group. Only 400 mg/kg of nano-copper induced the production of oxidative stress in hepatocytes of rats.

The levels of liver oxidative stress are shown in Figure 4. Levels of T-AOC were significantly induced by 200 and 400 mg/kg nano-copper. The levels of MDA, iNOS and NO increased significantly in 400 mg/kg nano-copper treated rats. The levels of superoxide dismutase (SOD) and cyclooxygenase (COX)-2 were increased in nano- and micro-copper treated rats, but there was no significant difference relative to control.

### 2.5. Signaling Pathway

**Figure 5:**
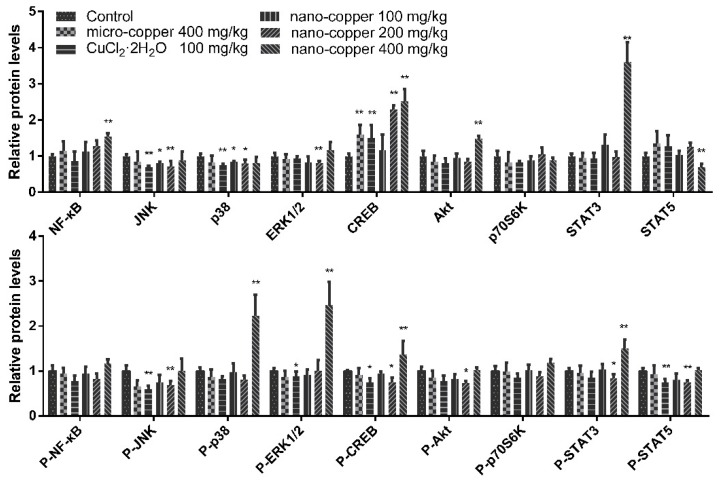
Changes in select signaling pathways in rat livers. * *p* < 0.05, ** *p* < 0.01 vs. control group. The P- showed the level of phosphorylated protein. The total signal proteins of NF-κB, CREB, Akt and STAT3, and the phosphorylated signal proteins of p-38, p-ERK1/2, CREB and STAT3 were induced by nano-copper.

Changes in the expression of marker proteins for various signaling pathways are illustrated in Figure 5. We studied the NF-κB, MAPK and STAT3 signaling pathways due to their known roles in CYP450 expression. Micro- and ion copper had little effect on the activation of these pathways. Nano-copper increased the total expression of NF-κB, cAMP-response element binding protein (CREB), Protein kinase B (Akt) and STAT3, but suppressed the expression of MAPK and STAT5. High-dose nano-copper activated signaling via phosphorylation of p-38, CREB and STAT3, but at the same time down-regulated expression of c-Jun N-terminal kinases (JNK) and STAT5. All the data were normalized to control.

### 2.6. Nuclear Receptors

**Figure 6:**
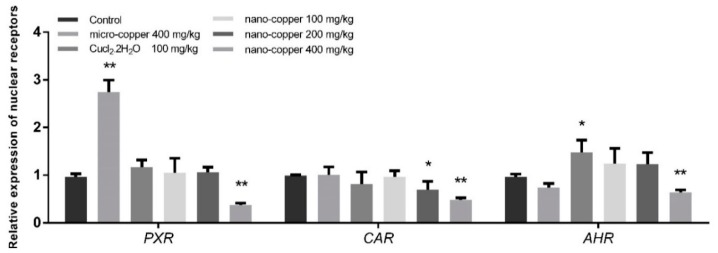
The mRNA levels of nuclear receptors in rat livers. * *p* < 0.05, ** *p* < 0.01 vs. control group. Nano-copper decreased the expression of *PXR*, *AHR* and *CAR*, but Micro-copper and CuCl_2_ increased the level of *PXR* and *AHR*, respectively.

The liver mRNA expression levels of *PXR* and *AHR* were significantly induced by Cu ion, though suppressed by 400 mg/kg nano-copper (Figure 6). The mRNA levels of *CAR* were suppressed by 200 and 400 mg/kg nano-copper.

### 2.7. CYP450 mRNA Expression

**Figure 7:**
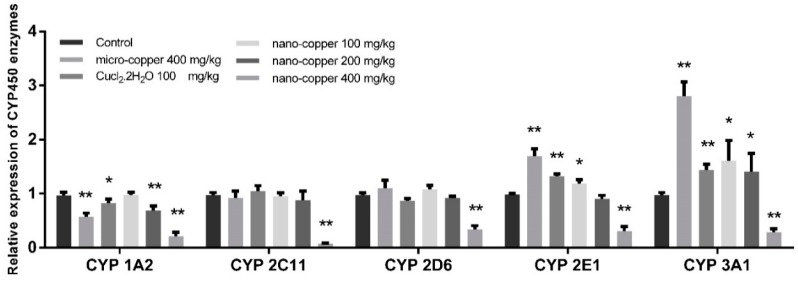
The mRNA levels of CYP450 enzymes in rat livers. * *p* < 0.05, ** *p* < 0.01. The expression of CYP450 enzymes were inhibited by nano-coper (200 mg/kg), but CYP 3A1 and CYP 2E1 were induced by Micro-copper, CuCl_2_ and low doses of nano-copper.

The mRNA expression levels of CYP450 enzymes are shown in Figure 7. The level of CYP 1A2 decreased significantly in micro-copper, Cu ion, and 200 and 400 mg/kg nano-copper treated rats. The levels of CYP 2C11 and 2D6 decreased significantly in 400 mg/kg nano-copper treated rats. The levels of CYP 2E1 and CYP 3A1 were increased after micro-copper, Cu ion, and 100 mg/kg nano-copper treatment, but suppressed by 400 mg/kg nano-copper treatment.

### 2.8. Activity Analysis

**Figure 8:**
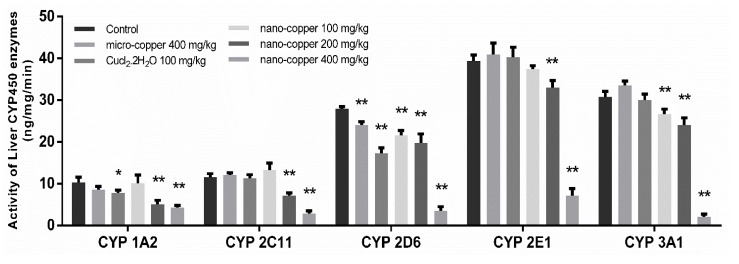
The activity of CYP450 enzymes. * *p* < 0.05, ** *p* < 0.01 vs. control group. The activity of CYP450 is inhibited by nano-copper in a dose-dependent manner.

The activities of CYP450 enzyme are shown in Figure 8. The activity of CYP 1A2 decreased significantly in Cu ion, 200 and 400 mg/kg nano-copper treated rats. The activity of CYP 2C11 and 2E1 decreased significantly in 200 and 400 mg/kg nano-copper treated rats. The activity of CYP 2D6 decreased significantly in copper treated rats, and 3A1 decreased significantly in three nano-copper treated rats.

## 3. Discussion

Nanoparticles are widely used in biotechnology and biomedical science due to their nanoscale and large surface area [33,34,35], but they have been found to sometimes produce toxicity. Biological safety and mechanism research into nano-copper is in its preliminary stages, and results thus far indicate potential toxicity for both humans and ecological systems [36]. In fact, copper is maintained in homeostasis in the human body, but an overload of copper in vivo can result in organ toxicity [37].

Studies have shown that nanoparticles can easily enter the bloodstream through the gastrointestinal system [6,7]. Gastrointestinal contact with nanoparticles is one of the most common exposure routes for humans. In mice, nano-gold particles induce greater toxicity when introduced via the oral route than via tail vein injection [38]. Unlike micro-copper, the same dose of nano-copper is more likely to accumulate in the liver and induce strong liver injury [39]. Nano-copper particles are able to interact with biomolecules due to their large specific surface area and their high reactive activity [40]. There are many examples of biochemical reactions that occur in cells which result in superoxide radical (O^2−^) production, leading to reactive oxygen species (ROS) accumulation and therefore oxidative stress [41]. ROS are oxygen derivatives that are highly reactive and tend to form adducts with DNA and proteins; examples include superoxide anions (O^2−^), hydroxyl radicals, and hydrogen peroxide. Modification of the redox state by nano- and microparticles has been reported as one of the main mechanisms of cytotoxicity at the cellular level [42,43]. In our results, the levels of iNOS, NO and MDA were increased significantly in rats treated with 400 mg/kg nano-copper, but micro- and ion-copper were found to have no effect on oxidative stress in rats. The levels of these molecules are generally considered a reliable index of oxidative stress. Although nano-copper did not increase the levels of all inflammatory cytokines, we found the levels of IL-6, IFN-γ, and MIP-1 increased significantly in 400 mg/kg nano-copper treated rat livers. Micro-copper also increased the expression of IL-6, TNF-α and IFN-γ, and all the copper particles decreased the level of IL-4 in the livers of rats. It has been found that MIP-1 is produced by macrophages after stimulation by nano-particles, which in turn induces the synthesis and release of other pro-inflammatory cytokines such as IL-1β, IL-6 and TNF-α from fibroblasts and macrophages [32]. White blood cells such as macrophages and neutrophils release high concentrations of ROS as a defensive measure in response to nanoparticle detection. However, nano-particle exposure-mediated oxidative stress leads to activation of STAT3, MAPK, Akt, and NF-κB, contributing to the pro-inflammatory cascade [44,45]. In our results, expression levels and phosphorylation of MAPK (p38 and ERK) and STAT3 were increased significantly by high-dose nano-copper. These changes are closely related to the regulation of CYP450 enzymes in the liver [46,47]. Therefore, we speculate that nano-copper is very likely to lead to drug metabolism enzyme disorders, and thus affect drug metabolism.

Drug-drug interaction (DDI) refers to the phenomenon where one drug’s effects on metabolism influences the pharmacokinetics of another drug. The inhibition and induction of CYP450 enzymes are the key mechanisms of DDIs [48]. Pharmacokinetic DDIs are responsible for approximately 20–30% of the adverse drug reactions experienced by patients, usually due to reduced drug efficacy or increased drug toxicity [49,50,51]. The majority of drugs are metabolized by the family CYP450 1, 2 and 3 in the liver, CYP450 3A4 being the main form metabolizing the largest proportion of drugs in humans [29]. In our results, the mRNA expression of CYP450 enzymes decreased significantly in 400 mg/kg nano-copper treated rats. The mRNA levels of CYP1A2 were also suppressed by micro-copper, Cu ion and 200 mg/kg nano-copper. The levels of CYP2E1 and CYP3A1 were induced by micro-copper, Cu ion, and 100 mg/kg nano-copper. This demonstrates that the CYP450 disruption of nano-copper was dose-dependent. A high dose of nano-copper decreased the mRNA expression of CYP450, but a low dose of nano-copper induced some CYP450 expression in the liver.

The following transcription factors are key to the regulation of genes coding for CYP450s and drug transporters in a wide variety of cell types: aryl hydrocarbon receptor (AHR), constitutive androstane receptor (CAR), and pregnane X receptor (PXR) [52]. Although PXR and CAR can independently regulate the expression of CYP450s, the regulating function is both specific and cross-talking [53]. PXR strongly binds to the DR4, DR3 or ER6 motif in both *CYP 2B6* and *CYP 3A4* promoters, and CAR only weakly binds to the *CYP 3A4* proximal ER6 motif but strongly binds to the imperfect DR4 motif in the phenobarbital-responsive enhancer module of the *CYP 2B6* gene [54,55,56], leading to CAR’s selectivity for CYP2B6 over CYP3A4 in the liver. This AHR complex binds to a specific DNA sequence called the dioxin or xenobiotic responsive element (DRE or XRE), inducing recruitment of transcriptional machinery to nearby promotors to promote gene expression. While AHR is involved in the promotion of a large array of genes, its prototypic target gene is *Cyp1A1* [57]. Pro-inflammatory cytokines, such as IL-1β, IL-4, IL-6, TNF-α and IFN-γ downregulate major CYP enzymes with specific effects on mRNA levels, protein expression, and enzyme activity [58]. Oxidative stress can indirectly affect the expression of CYP450 mRNA by affecting the expression of nuclear receptors [59]. The mRNA expression of CYP450 enzymes decreased in 400 mg/kg nano-copper treated rats, which may have been caused by the increased levels of inflammation and oxidative stress and decreased levels of PXR, CAR and AHR. Nano-copper does affect IκBα/NF-κB, MAPKs nor mitochondrial signaling [60,61], and most of the signaling pathways play a significant role in the downregulation of CYP450 enzymes [62]. In our results, nano-copper caused liver injury via increased inflammatory cytokine production and oxidative stress. Nano-copper also activated the NF-κB signal pathway, which regulates CYP450 mRNA expression and activity by mutual repression with some nuclear receptors [63]. Nano-copper also activated the MAPK (p-38 and ERK), CREB and STAT3 signaling pathways, but how these signaling pathways act on the regulation of CYP450s requires further study. The change of the mRNA expression and activity levels of CYP450 enzymes was consistent, but the activity of CYP450s decreased more obviously, indicating that the nano-copper affected both transcription and translation of CYP450 enzymes. The influence of micro-copper and copper ions on CYP450 is obviously different from that of nano-copper. Micro-copper does not induce severe oxidative stress in the liver like nano-copper does, and therefore has less influence on downstream signaling pathways and nuclear receptor expression. Therefore, the particle size is the main factor causing hepatic toxicity and CYP450 disruption in nano-copper treatment [64,65]. Nano-sized copper particles have been demonstrated by our research to cause more serious liver dysfunction.

## 4. Materials and Methods

### 4.1. Test Chemicals and Preparation of Test Chemicals

Nano-copper (H1605061; 99.8% purity), micro-copper (A1711069; 99.6% purity) and CuCl_2_·2H_2_O (F1620012; 99.8% purity) were purchased from Shanghai Aladdin biochemical technology co. LTD (Shanghai, China). The particle size (measured by TEM, Hitachi, Tokyo, Japan) of nano-copper and micro-copper was 80 nm and 1 μm, respectively. Hydroxypropylmethylcellulose (HPMC) suspension vehicle was purchased from Sigma-Aldrich (St. Louis, MO, US). All other chemicals were of the analyze grade commercially available. Test nano-copper were dispersed into 1% HPMC solution (*w*/*v*) with water. The suspensions were made before use and prepared by ultrasonic dispersion (FS30D, Fisher Scientific, Hampton, MA, USA) on ice for 20 min (130 W, 20 kHz).

### 4.2. Particle Characterization

The size of nano- and micro-coppers was characterized with a scanning electron microscope (Phenom ProX, Nani Scientific Instruments LTD, Shanghai, China). The nano-coppers were added to purified water to obtain a stock suspension of 10 g/L, which was then shaken and sonicated (40 Hz) for 30 min in a sonication ice bath. The distribution of particle sizes in this suspension, as indicated by its polydispersity index, was characterized during dynamic light scattering studies performed with a Zeta sizer Nano ZS (Malvern Instruments, Malvern, UK) immediately after sonication.

### 4.3. Experimental Protocols and Dose Selection

This study was reviewed and approved by the Animal Ethical Committee of Sichuan Agricultural University (No. 20170314, 14 March 2017). As males are more susceptible to the toxic effects of nano-copper than females [9,66], we utilized male Sprague-Dawley rats for the in vivo toxicity study. A total of 60 healthy male rats (body weight: 180–220 g) were randomly assigned to six experimental groups (*n* = 10). Nano-copper was administered by oral gavage to rats at doses of 100, 200, and 400 mg/kg/day, and three control groups were given 400 mg/kg/day micro-copper, 100 mg/kg/day Cu ion (CuCl_2_·2H_2_O) and vehicle 1% HPMC alone, respectively. The concentrations were 80 (400 mg/kg and micro-copper), 40 (200 mg/kg) and 20 mg/mL (100 mg/kg and Cu ion) in 1% HPMC, the administration volume (5 mL/kg body weight) of nano-copper and micro-copper was calculated based on the body weight of the individual animal. The experimental doses were selected based on the results of a preliminary acute toxicity study and clinical dose of feed. Acute toxicity experiments were performed to determine the LD_50_ (median lethal dose) values of copper nano-particles, micro-particles, and ions. The rats were administered a single oral gavage dose of nano-coppers, Cu micro-particles or Cu ions at levels recommended by the organization for economic co-operation and development (OECD). Individual animals were dosed in a specific sequence. The first animal received a dose one step below the estimated LD_50_ dose. Dose was increased if the animal survived and reduced if the animal died. Copper micro-particles were found to be nontoxic when administered at doses below the regulatory limit, i.e., 5000 mg/kg, hence, they were evaluated at a dose of 5000 mg/kg. The LD_50_ and 95% profile likelihood values for the copper nano-particles, micro-particles, and ions were obtained by analyzing experimental data with the AOT_425_ StatPgm software program. The results showed that the LD_50_ values of Cu ions, nano-copper, and micro-copper particles were 359.6 mg/kg, 2075 mg/kg, and >5000 mg/kg, respectively. At 400 mg/kg of the nano-copper group, the rats displayed obvious general toxicity, such as slowly body weight gain, decreased food intake, and mental depression. Based on these results, 400 mg/kg/day was used as the highest-dose, and middle- and low doses were 200 and 100 mg/kg, respectively. The dose levels of micro-copper were only selected as the highest dose of 400 mg/kg for comparing the toxic effects. All animals were observed twice daily for any clinical signs of toxicity and mortality throughout the whole study period. The body weight of each rat was measured prior to the beginning of treatment and after experimentation. During exposure to the test agents, a maximum of 5 animals were housed in each cage, and food and water were available ad libitum. The animals were killed 24 h (test day 7) after the final administration of different coppers.

### 4.4. Sample Collection

After an overnight 8-h fasting period, all animals in the six groups were anesthetized with isoflurane, and the livers were quickly separated. The liver microsomes were prepared to analyze the activity of cytochrome P450 enzymes. The levels of cytokines and antioxidant levels were analyzed in 1 g liver tissue. 2 g samples of liver tissue were stored in liquid nitrogen for later use in mRNA and gene expression analyses. The remaining samples of liver tissue were fixed in 10% formalin for histologic examination. Liver microsomes were prepared by differential centrifugation [67]. Briefly, the liver of each rat was excised, rinsed with ice-cold saline (0.9% NaCl *w*/*v*), weighed, and homogenized in 0.05mM Tris/KCl buffer (pH 7.4). The homogenate was centrifuged at 10,000× *g* at 4 °C for 30 min, after which, the resultant supernatant fraction was centrifuged at 105,000× *g* at 4 °C for 60 min. The pellet was suspended in 0.05 mM Tris/KCl buffer (pH 7.4) and stored at −80 °C until use. The liver microsome protein concentrations were determined by use of a Bradford protein assay kit (Tiangen Biotech Co LTD; Beijing, China).

### 4.5. Histology

The histopathological evaluation was performed after the liver tissue was fixed in a 10% neutral-buffered formalin solution for 1 week. The tissues were stained with hematoxylin and eosin for further examination.

### 4.6. Measurements of Cytokines

Liver tissue were added to cold saline (0.9% NaCl) at a ratio of 1:4, homogenized in an ice bath, then centrifuged (3000× *g*, 10 min) to take the supernatant for testing. The levels of interleukin (IL)-1β, IL-2, IL-4, IL-6, tumor necrosis factor-alpha (TNF-α), monocyte chemotactic protein-1 (MCP-1) and macrophage inflammatory protein (MIP)-1α were analyzed according to the manufacturer’s instructions (R&D Systems).

### 4.7. Measurements of Oxidative Stress

The levels of oxidative stress were evaluated through the measurement of total antioxidant capacity (T-AOC), catalase (CAT), superoxide dismutase (SOD), glutathione peroxidase (GSH-Px), nitric oxide synthase (iNOS), cyclooxygenase-2 (COX-2), and the contents of malondialdehyde (MDA), and nitric oxide (NO) in the livers of rats in accordance with the kits’ instructions (Nanjing Jiancheng Bioengineering Institute, Nanjing, China).

### 4.8. Measurements of the Signaling Pathway

Liver tissue homogenate was collected in Eppendorf tubes and was allowed to clot at room temperature for 1 h before centrifugation at 10,000× *g* for 10 min at 4 °C. The protein levels were measured by multiplexed particle-based flow cytometric cytokine assay kits, as described previously [68,69]. Luminex technology was used according to the manufacturer’s instructions to measure protein and phosphoproteins of CERB, NF-κB, P38, ERK1/2, Akt, p70s6k, STAT3 and STAT5 in the livers of the rats (R&D Systems).

### 4.9. RNA Extraction and Determination of Gene Expression in Liver by Real-Time PCR

The gene expression of liver was performed as previously described [70]. The quality and quantity of extracted mRNA were determined with UV spectroscopy (NanoDrop 2000 UV-Vis Spectrophotometer, Thermo Scientific; Waltham, MA, USA). The following target genes were analyzed for their expression: *constitutive androstane receptor (CAR)*, *pregnane x receptor (PXR)*, *Aryl hydrocarbon receptor* (*AHR*), *CYP1A2*, *2C11*, *2D6*, *2E1,* and *3A2*. *GAPDH* was used as a housekeeping gene for data normalization (Table 1).

### 4.10. Measurements of CYP1A2, 2C11, 2D6, 2E1 and 3A1 Activity

The activity of *CYP1A2*, *2C11, 2D6, 2E1* and *3A2* was assessed as previously described [71,72]. The microsomal incubations were conducted for 60 min at 37 °C in a final volume of 500 μL. Each incubation mixture contained microsomes (1.0 mg protein/mL) and an NADPH-regenerating system consisting of G-6-P (10 mM), PDH (2.0 U/mL), MgCl_2_ (10 mM) and NADP^+^ (1.0 mM). 10μL of acetonitrile was added to each pool, which contained five probe substrates for different specific CYP450 enzymes. The activities of the five CYP450 enzymes were evaluated based on reduction of five probe substrates: phenacetin for CYP1A2, tolbutamide for CYP2C11, dextromethorphan for CYP2D6, chlorzoxazone for CYP2E1, and testosterone for CYP3A2. The concentrations of phenacetin, tolbutamide, dextromethorphan, chlorzoxazone and testosterone in the incubation mixtures were 100, 100, 100, 100, and 800 μg/mL, respectively. All incubations were terminated by adding 500 μL of ice-cold acetonitrile containing 20 ng/mL tinidazole (IS), after which, the solutions were thoroughly mixed and centrifuged (18,000× *g* at 4 °C for 10min) to obtain the supernatant fractions, of which 10 μL was used for the cocktail HPLC analysis.

**Figure 9:**
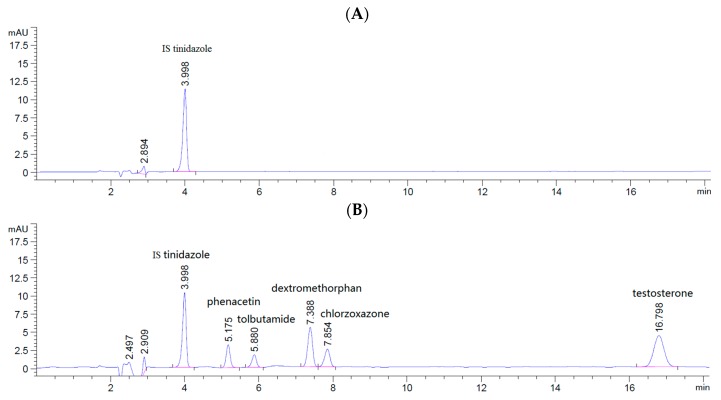
Chromatograms of five cocktail probe drugs and tinidazole (IS) in the incubation system. IS in blank sample (**A**); sample spiked with five cocktail probe drugs and IS (**B**).

Phenacetin, tolbutamide, dextromethorphan, chlorzoxazone, testosterone and tinidazole (IS) were analyzed by use of a 1260 series HPLC instrument (Agilent Technologies; Santa Clara, CA, USA) capable of diode column detector detection at 230 nm. HPLC was performed at room temperature with an Agilent reverse-phase C18 column (Zorbax SB-C18, 4.6 × 250 mm, 5 μm, Agilent, Santa Clara, CA, US) equipped with a C18 guard column. The mobile phase for HPLC consisted of acetonitrile and 0.01 M acetic acid water at the ratio of 4:6; the flow rate was 1.0 mL/min. IS tinidazole (IS), phenacetin, tolbutamide, dextromethorphan, chlorzoxazone and testosterone eluted at 3.998, 5.175, 5.880, 7.388, 7.854 and 16.789 min, respectively (Figure 9). The regression equations and lower limit of quantitation (LLOQ) concentrations for the analytes are shown in Table 2.

### 4.11. Statistical Analysis

The numerical data are presented as means ± standard deviations (SD), and all statistical comparisons were analyzed by one-way analysis of variance (ANOVA) followed by Dunnett’s multiple comparison test. A *p* value of <0.01 and 0.05 were considered significant compared with control group. Statistical analyses were performed using the GraphPad InStat v.3.0 (GraphPad Software, Inc., La Jolla, CA, USA).

## 5. Conclusions

In summary, we described the in vivo toxicity and mechanism on liver CYP450 enzymes of nano-copper following repeated oral exposure. We showed that high-dose nano-copper affects the oxidant-antioxidant balance and induced inflammation in rat livers, resulting in the significant decrease of CYP450 enzyme expression. The suppressive effect on CYP450 is mainly in relation to the low expression of PXR, CAR and AHR, and also in relation to the activation of the MAPK (p-38 and ERK), CREB and STAT3 signaling pathways. Although high-dose nano-copper inhibited the mRNA expression of CYP450s, Cu ion, micro-copper and low-dose nano-copper (100 mg/kg) induced the mRNA expression of CYP2E1 and CYP3A1. Explanation of this phenomenon requires further study. Under these experimental conditions, nano-copper exerted adverse effects on CYP450 enzymes when the dose was 400 mg/kg/day in rats. In light of our findings, when nano-copper is used as a pharmaceutical or animal feed additive, attention should be paid to its potential impact on drug metabolism and drug-drug interactions.

## Figures and Tables

**Table 1:** PCR primers used for analysis of gene expression.

Target	Sequences of Primers (5′ to 3′)	Base Number
*CYP1A2* F	GGTGGAATCGGTGGCTAAT	19
*CYP1A2* R	AGTCCTTGCTGCTCTTCACG	20
*CYP2C11* F	AATCCGCAGTCTGAGTTTACCC	22
*CYP2C11* R	GGTTTCTGCCAATTACACGTTCT	23
*CYP2D6* F	AGCTTCAACACCGCTATGGT	20
*CYP2D6* R	CAGCAGTGTCCTCTCCATGA	20
*CYP2E1* F	CCTTTCCCTCTTCCCATCC	19
*CYP2E1* R	AACCTCCGCACATCCTTCC	19
*CYP3A1* F	TGCCATCACGGACACAGA	18
*CYP3A1* R	ATCTCTTCCACTCCTCATCCTTAG	24
*PXR* F	GACGGCAGCATCTGGAACTAC	21
*PXR* R	TGATGACGCCCTTGAACATG	20
*CAR* F	CCACGGGCTATCATTTCCAT	20
*CAR* R	CCCAGCAAACGGACAGATG	19
*AHR* F	TGGACAAACTCTCCGTTCTAAGG	23
*AHR* R	GATTTTAATGCAACATCAAAGAAGCT	26
*GAPDH* F	GATGGTGAAGGTCGGTGTG	19
*GAPDH* R	ATGAAGGGGTCGTTGATGG	19

**Table 2:** Regression equation, linear range, and LLOQ for the probe substrates used in incubations.

Analytes	Regression Equation	Correlation Coefficient (*R*^2^)	Linear Range (ng/mL)	LLOQ (ng/mL)
Phenacetin	*y* = 166.9*x* + 1.3728	0.9995	200–1400	50
Tolbutamide	*y* = 176.97*x* + 0.6415	0.9996	200–1400	50
dextromethorphan	*y* = 128.91*x* + 1.3531	0.9999	200–1400	50
chlorzoxazone	*y* = 110.85*x* + 4.8935	0.9999	200–1400	50
Testosterone	*y* = 212.16*x* + 49.721	0.9994	800–11,200	100
